# β‐Cell glucokinase expression was increased in type 2 diabetes subjects with better glycemic control

**DOI:** 10.1111/1753-0407.13380

**Published:** 2023-03-20

**Authors:** Jingwen Liu, Hui Fu, Fuyun Kang, Guang Ning, Qicheng Ni, Weiqing Wang, Qidi Wang

**Affiliations:** ^1^ Department of Endocrine and Metabolic Diseases, Shanghai Institute of Endocrine and Metabolic Diseases, Ruijin Hospital Shanghai Jiao Tong University School of Medicine Shanghai China; ^2^ Shanghai National Clinical Research Center for Metabolic Diseases, Key Laboratory for Endocrine and Metabolic Diseases of the National Health Commission of the PR China Shanghai Jiao Tong University School of Medicine Shanghai China; ^3^ Shanghai Key Laboratory for Endocrine Tumor, State Key Laboratory of Medical Genomics, Ruijin Hospital Shanghai Jiao Tong University School of Medicine Shanghai China; ^4^ Sino‐French Research Center for Life Sciences and Genomics Ruijin Hospital Affiliated to Shanghai Jiao Tong University School of Medicine Shanghai China

**Keywords:** beta cell, glucokinase, glucokinase activators, type 2 diabetes, unfolded protein response, β细胞, 葡萄糖激酶, 葡萄糖激酶激活剂, 2型糖尿病, 未折叠蛋白反应

## Abstract

**Background:**

Type 2 diabetes (T2D) is characterized by a progressive deterioration of β‐cell function with a continuous decline in insulin secretion. Glucokinase (GCK) facilitates the rate‐limiting step of glycolysis in pancreatic β‐cells, to acquire the proper glucose‐stimulated insulin secretion. Multiple glucokinase activators (GKAs) have been developed and clinically tested. However, the dynamic change of human pancreatic GCK expression during T2D progression has not been investigated.

**Methods:**

We evaluated GCK expression by measuring the average immunoreactivity of GCK in insulin^+^ or glucagon^+^ cells from pancreatic sections of 11 nondiabetic subjects (ND), 10 subjects with impaired fasting glucose (IFG), 9 with well‐controlled T2D (wT2D), and 5 individuals with poorly controlled T2D (uT2D). We also assessed the relationship between GCK expression and adaptive unfolded protein response (UPR) in human diabetic β‐cells.

**Results:**

We did not detect changes of GCK expression in IFG islets. However, we found β‐cell GCK levels were significantly increased in T2D with adequate glucose control (wT2D) but not in T2D with poor glucose control (uT2D). Furthermore, there was a strong positive correlation between GCK expression and adaptive UPR (spliced X‐box binding protein 1 [XBP1s] and activating transcription factor 4 [ATF4]), as well as functional maturity marker (urocortin‐3 [UCN3]) in human diabetic β‐cells.

**Conclusions:**

Our study demonstrates that inductions of GCK enhanced adaptive UPR and UCN3 in human β‐cells, which might be an adaptive mechanism during T2D progression. This finding provides a rationale for exploring novel molecules that activate β‐cell GCK and thereby improve pharmacological treatment of T2D.

## Graphical abstract

**Highlights**

- A significant upregulation of glucokinase (GCK) expression was found in type 2 diabetes (T2D) subjects with adequate glucose control, but such increase was undetected in T2D with poor glycemic control.
- A strong positive correlation between GCK levels and spliced X‐box binding protein 1 (XBP1s)/activating transcription factor 4 (ATF4) expressions was observed in β‐cells from T2D patients.
- Successful GCK activation to induce adaptive unfolded protein response in human β‐cells might be an adaptive mechanism for maintaining appropriate glycemia.

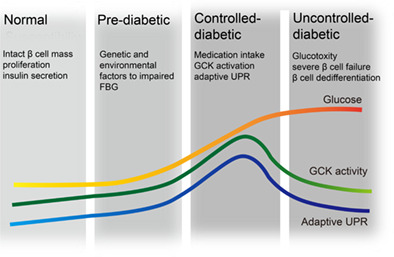

## 摘要

**背景:**2型糖尿病(T2D)以胰岛β细胞功能进行性恶化和胰岛素分泌持续下降为特征。葡萄糖激酶(GCK)促进胰岛β细胞糖酵解的限速步骤, 以获得适当的葡萄糖刺激的胰岛素分泌。多种葡萄糖激酶激活剂(GKA)已经被研发出来并进行了临床测试, 但在T2D进展过程中, 人胰腺GCK表达的动态变化尚未被研究。

**方法:**我们通过测定11名非糖尿病受试者(ND)、10名空腹血糖受损(IFG)受试者、9名血糖控制良好的2型糖尿病患者和5名血糖控制不佳的2型糖尿病患者胰腺切片中胰岛素细胞或胰高血糖素分泌细胞中GCK的平均免疫反应性来评估GCK的表达。我们还评估了GCK表达与糖尿病患者β细胞适应性未折叠蛋白反应(UPR)之间的关系。

**结果:**我们没有检测到空腹血糖受损患者胰岛中GCK表达的变化, 但是我们发现β细胞GCK水平在血糖控制良好的T2D (wT2D)中显著增加, 而在血糖控制不佳的T2D (uT2D)中却没有。此外, 在糖尿病患者β细胞中, GCK的表达与适应性UPR (XBP1s和ATF4)以及功能性成熟标记物(UCN3)呈强正相关。

**结论:**我们的研究表明, GCK的诱导增强了人β细胞的适应性UPR和UCN3, 这可能是T2D进展中的一种适应性机制。这一发现为探索激活β细胞GCK的新分子以及改善T2D的药物治疗提供了理论基础。

## 1 INTRODUCTION

Type 2 diabetes (T2D) is characterized by a progressive deterioration of β‐cell function with a continuous decline in insulin secretion.^1^ β‐cell displays a remarkable metabolic specialization, such as expressing glucokinase (GCK) enzyme, which catalyzes the rate‐limiting step of glycolysis, to acquire the proper glucose‐stimulated insulin secretion.^2^ Patients with mutation in the GCK are known as maturity‐onset diabetes of the young, manifested as permanent and early onset of hyperglycemia if both alleles are affected.^3^ Activation of GCK by glucokinase activators (GKAs) presented an alternative approach to improve glycemic control by either stimulating insulin release from pancreatic β‐cells or promoting hepatic glucose uptake and glycogen synthesis in the liver.^4^, ^5^, ^6^, ^7^, ^8^ In fact, multiple GKAs have been developed and clinically tested, but many of them were discontinued due to lack of long‐term efficacy and induction of hypoglycemia and/or dyslipidemia.^6^, ^9^ A novel dual‐acting GKA, Dorzagliatin, improved glycemic control and β‐cell function in patients with T2D during 52‐week treatment.^7^, ^10^ The potential of GKAs in diabetes therapy spurs us to explore the pathophysiological role of GCK on human pancreatic β‐cell during T2D progression.

GCK plays an important role in regulating functional β‐cell mass, and its reduced expression has been suggested to involve the pathogenesis of T2D.^11^ It has been reported that activation of GCK has proliferative and antiapoptotic effects on pancreatic β‐cells in in vitro and in vivo models.^12^, ^13^, ^14^ On the other hand, long‐term activation of GCK in β‐cell specific gain‐of‐function mutant GCK mice resulted in β‐cell failure through increased endoplasmic reticulum (ER) stress and DNA damage.^15^, ^16^ Kazuno Omori et al reported that reducing GCK activity enhanced insulin secretion, preserved β‐cell mass and decreased metabolic stress‐related genes in islets of db/db mice.^17^ There is currently very limited reliable knowledge of dynamic GCK expression in human islets during T2D progression. It is also unknown whether the excess glycolysis upon GCK activation in prediabetic and diabetic islets might induce β‐cell overexhaustion and affect their consequent function.

In the present study, we evaluated GCK expression by measuring the average immunoreactivity of GCK in insulin^+^ or glucagon^+^ cells from pancreatic sections of nondiabetic (ND), impaired fasting glucose (IFG), and T2D patients with different glycemic control. We did not find an induction of GCK expression in IFG islets. However, we found β‐cell GCK levels were significantly increased in T2D with adequate glucose control (wT2D) but not in T2D with poor glucose control (uT2D). Furthermore, there was a strong positive correlation between GCK expression and adaptive unfolded protein response (UPR) (X‐box binding protein 1 [XBP1s] and activating transcription factor 4 [ATF4]), as well as functional maturity marker (urocortin‐3 [UCN3]) in human diabetic β‐cells. These data suggest that activation of GCK might serve as one of the adaptation mechanisms in human β‐cells, which benefits the glycemic control in T2D patients. Our study provides clues supporting β‐cell GCK as a promising therapeutic target for human type 2 diabetes.

## 2 MATERIALS AND METHODS

### 2.1 Patients and Human Pancreas

A total of 2558 partial pancreatectomy cases for various reasons performed in the Department of Surgery in Ruijin Hospital between January 2013 and September 2020 were obtained. The data set includes electronic medical records and pathology diagnosis. Case subjects were identified based on these preferences in the Department of Pathology, Ruijin Hospital database. We excluded those who had been reported as having pancreatitis and carrying a malignant tumor by reviewing their pathology diagnosis. Classification of normal glucose tolerance, prediabetes, or diabetes was performed according to clinical history and fasting blood glucose (FBG) according to criteria of the American Diabetes Association.^18^ Fasting blood samples were collected before pancreatic surgery to assess metabolic traits. Participants were classified into three groups according to FBG levels: ND (FBG <5.6 mmol/L and no history of diabetes); IFG (FBG 5.6 to ≤7.0 mmol/L and no history of diabetes); and T2D (FBG >7.0 mmol/L or a history of diabetes). We further divided T2D individuals into wT2D and uT2D subgroups, taking a cutoff of FBG as 8.1 mmol/L. Altogether, 11 cases of ND, 10 cases of IFG, 9 cases of wT2D, and 8 cases of uT2D (amid which 3 cases with undesirable pathologic sections exhibited no islets and therefore were excluded) were included in the present study. The paraffin sections of pancreas far from the margin of pancreatectomy were obtained from the Department of Pathology in Ruijin Hospital for subsequent analysis. All patients in the groups were age‐ and body mass index (BMI)‐matched. These pancreatic tissues were revalidated by the pathologist to ensure their pathology diagnosis. This study was approved by the Institutional Review Board of the Ruijin Hospital affiliated to Shanghai Jiao Tong University School of Medicine and was in accordance with the principles of the Declaration of Helsinki.

### 2.2 Immunofluorescence

A standard immunohistochemistry protocol was conducted for fluorescent immunodetection of various proteins in pancreatic sections as previously described.^19^ Pancreas was fixed in 4% paraformaldehyde and embedded in paraffin. Sections were deparaffinized and rehydrated. For antigen retrieval, slides were emerged in antigen unmasking solution (H‐3300, Vector) and placed in a pressure cooker for boiling. Slides were blocked with antibody diluent (DAKO) for 1 h at room temperature, followed by incubation in blocking solution with primary antibodies overnight at 4°C. The following primary antibodies were used: rabbit anti‐GCK (1:100, Proteintech Cat# 19666‐1‐AP, RRID: AB_10863656), guinea pig anti‐INSULIN (1:200, Abcam Cat# ab7842, RRID: AB_306130), mouse anti‐GLUCAGON (1:200, Abcam Cat# ab10988, RRID: AB_297642), rabbit anti‐XBP1s (ABclonal Cat# A17007, RRID: AB_2772919), rabbit anti‐ATF4 (1:200, Proteintech Cat# 10835‐1‐AP, RRID: AB_2058600) rabbit anti‐UCN3 (1:200, Sigma‐Aldrich Cat# HPA038281 RRID: AB_10672408), rabbit anti‐GADD34 (1:500, Abmart Cat#T61809 RRID: AB_2928981). Detection was performed by using Alexa Fluor 488 and 594 (1:500, Jackson ImmunoResearch Laboratories).

### 2.3 Image acquisition and analysis

Images were captured with a Zeiss LSM 880 confocal microscope. Image quantifications were performed blindly using ImageJ software (National Institutes of Health) with the EzColocalization plugin according to the protocol described by Stauffer et al.^20^ Briefly, quantifications of each islet's immunostaining intensity of GCK were measured on the positive area of insulin/glucagon using automatic plugin recognition, similarly does the immunostaining intensity of XBP1s, ATF4, and UCN3 on the INSULIN positive area. The number of islets evaluated for each analysis is presented on Table S2.

### 2.4 Statistical analysis

Data are presented as the mean ± SD or mean (minimum‐maximum). Statistical comparisons of the mean values among the groups were performed using one‐way analysis of variance with post hoc Bonferroni corrections or unpaired two‐tailed Student's *t* test for continuous variable. *p* values for the comparison of sex distribution between the groups were assessed using the Fisher's exact test. *p* values less than 0.05 were considered statistically significant. All statistical analyses were conducted using SPSS 17.0 (IBM).

## 3 RESULTS

### 3.1 Glucokinase expression in islets from ND, IFG, and T2D subjects

To evaluate the changes in glucokinase expression in human islets during T2D progression, we collected human pancreatic sections from 11 nondiabetic (ND, FBG: [4.50–5.47] mM), 10 impaired fasting glucose (IFG, FBG: [5.66–6.80] mM), 9 wT2D (FBG: [5.00–7.36] mM), and 5 uT2D (FBG: [8.15–13.7] mM) subjects. The detailed clinical characteristics of the investigated participants were summarized in Table 1 and Table S1. Age, sex, and BMI were matched in the four groups to exclude the confounding factors. IFG and T2D patients had significantly higher FBG levels compared to ND (6.1 ± 0.4 mM in IFG, 7.7 ± 2.8 in T2D vs 5.0 ± 0.3 in ND, *p* < 0.01, Table 1). For type 2 diabetes, uT2D individuals had significantly higher FBG (10.6 ± 2.9 vs 6.1 ± 0.8 mmol/L, *p* < 0 .001, Table 1) than wT2D.

**TABLE 1:** Clinical characteristics of the investigated participants.

Characteristic	ND	IFG	T2D			*p* value
			Overall	wT2D	uT2D	
*n*	11	10	14	9	5	
Diabetes history	No	No	Yes			
Age (years)	52.5 ± 14.4	56.6 ± 7.7	61.9 ± 9.0	58.9 ± 8.7	67.4 ± 7.1	0.11
Female sex, no. (%)	5 (45.5%)	5 (50.0%)	8 (57.1%)	6 (66.7%)	2 (40.0%)	0.77
BMI (kg/m^2^)	22.3 ± 2.4	24.1 ± 2.1	23.3 ± 2.6	23.7 ± 2.8	22.6 ± 2.5	0.24
FBG (mmol/L)	5.0 ± 0.3	6.1 ± 0.4	7.7 ± 2.8	6.1 ± 0.8	10.6 ± 2.9	0.003

*Note*: All values are expressed as mean ± SD. Analysis of variance with Bonferroni's post hoc test and Fisher's exact test were used for comparisons.

Abbreviations: BMI, body mass index; FBG, fasting blood glucose; IFG, impaired fasting glucose; ND, nondiabetes; uT2D, uncontrolled T2D; wT2D, well‐controlled type 2 diabetes.

**FIGURE 1:**
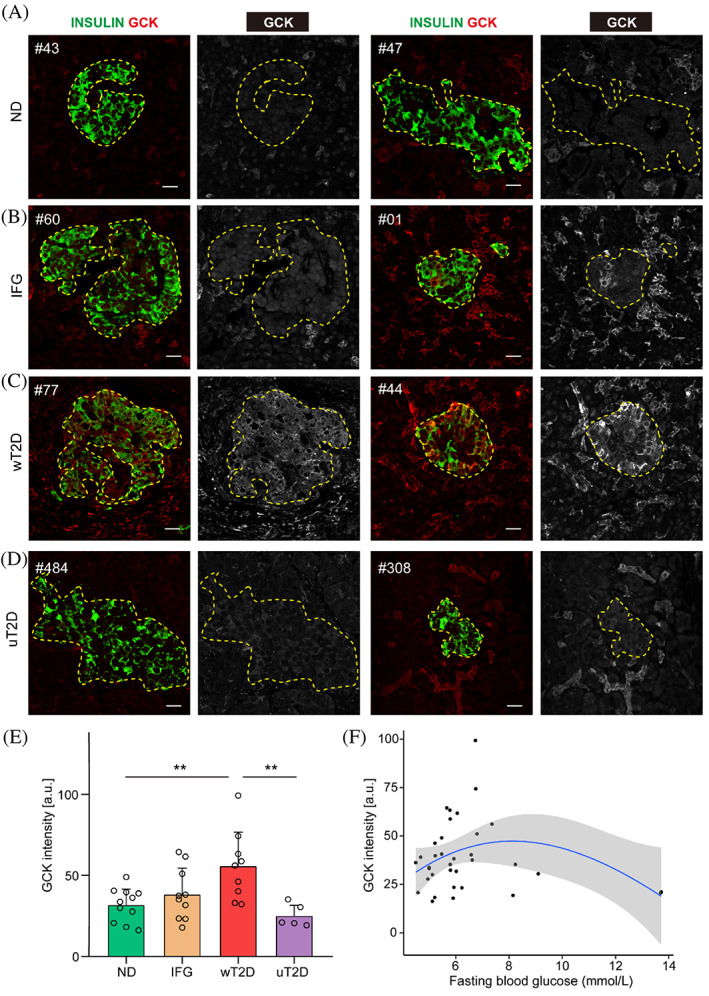
Dynamic changes of glucokinase (GCK) expression in human β‐cells during the progress of type 2 diabetes. (A–D) Representative images of pancreatic sections stained for GCK (red in merged channels and grayscale in split channel) from nondiabetic (ND; (A), cases No.43 and No.47), impaired fasting glucose (IFG; (B), cases No.60 and No.01), well‐controlled type 2 diabetes (wT2D; (C), cases No.77 and No.44), and uncontrolled type 2 diabetes (uT2D; (D), cases No.484 and No.308) individuals. Scale bars, 20 μm. (E) Quantificational and statistical analysis of mean GCK intensity of β‐cells in ND (*n* = 11), IFG (n = 10), wT2D (*n* = 9), and uT2D (*n* = 5). (F) Smooth line was fitted using loess method to explore the variation of GCK expression in β‐cells across the fasting blood glucose. Data presented as means ± SD. Analysis of variance with Bonferroni post hoc test was performed between the four groups. *p* less than 0.05 was considered statistically significant. **p < 0.01.

We performed double staining for GCK and INSULIN (Figure 1A–D) and further quantified the mean GCK fluorescent intensities in β‐cells of each individual (Figure 1E). Violin plots showed the distribution of average GCK intensity in each patient (Figure S1). Heterogeneity of GCK expression was observed in different islets of the same individual (Figure S1). Moreover, in IFG and T2D patients, there was a big variation of GCK expression in individuals within the same group (Figure S1).

GCK expression was nearly undetectable in ND islets (Figure 1A), and more INSULIN positive cells were recruited into elevated GCK activity in IFG and wT2D islets (Figure 1B,C). However, we did not detect significant differences in mean β‐cell GCK expression between IFG and ND subjects (Figure 1E). Importantly, the mean GCK expression from wT2D patients was significantly higher than that from ND participants (56.0 ± 21.5 vs 31.9 ± 10.4, *p* = 0.009, Figure 1E). But this was not the case in uT2D subjects: their mean GCK expression was only half the level of that in wT2D (25.3 ± 7.1 vs 56.0 ± 21.5, *p* = 0.007, Figure 1E), which was comparable to the levels in ND (Figure 1E). We also checked the GCK expression in α‐cells but did not detect significant changes in glucagon expressing cells among the four groups (Figure S2).

### 3.2 Glucose‐dependent biphasic change of β‐cell GCK expression in human

To examine the possible link between β‐cell GCK expression and the clinical parameters, we assessed correlation analysis between GCK levels and age, BMI, and FBG. There was no significant association between β‐cell GCK expression and age (*r* = 0.13, *p* = 0.46), BMI (*r* = 0.27, *p* = 0.12), and FBG (*r* = −0.14, *p* = 0.42) when all the subjects were examined. Interesting, by using a loess regression analysis, we found β‐cell GCK expression increased along with the rising fasting glucose, reached a peak at FBG around 8 mmol/L, and thereafter decreased when FBG further increased (Figure 1F). These results revealed a biphasic correlation of FBG with GCK expression, which relies on blood glucose level of the individuals (Figure 1F).

### 3.3 GCK was positively related with adaptive UPR response in diabetic human β‐cells

**FIGURE 2:**
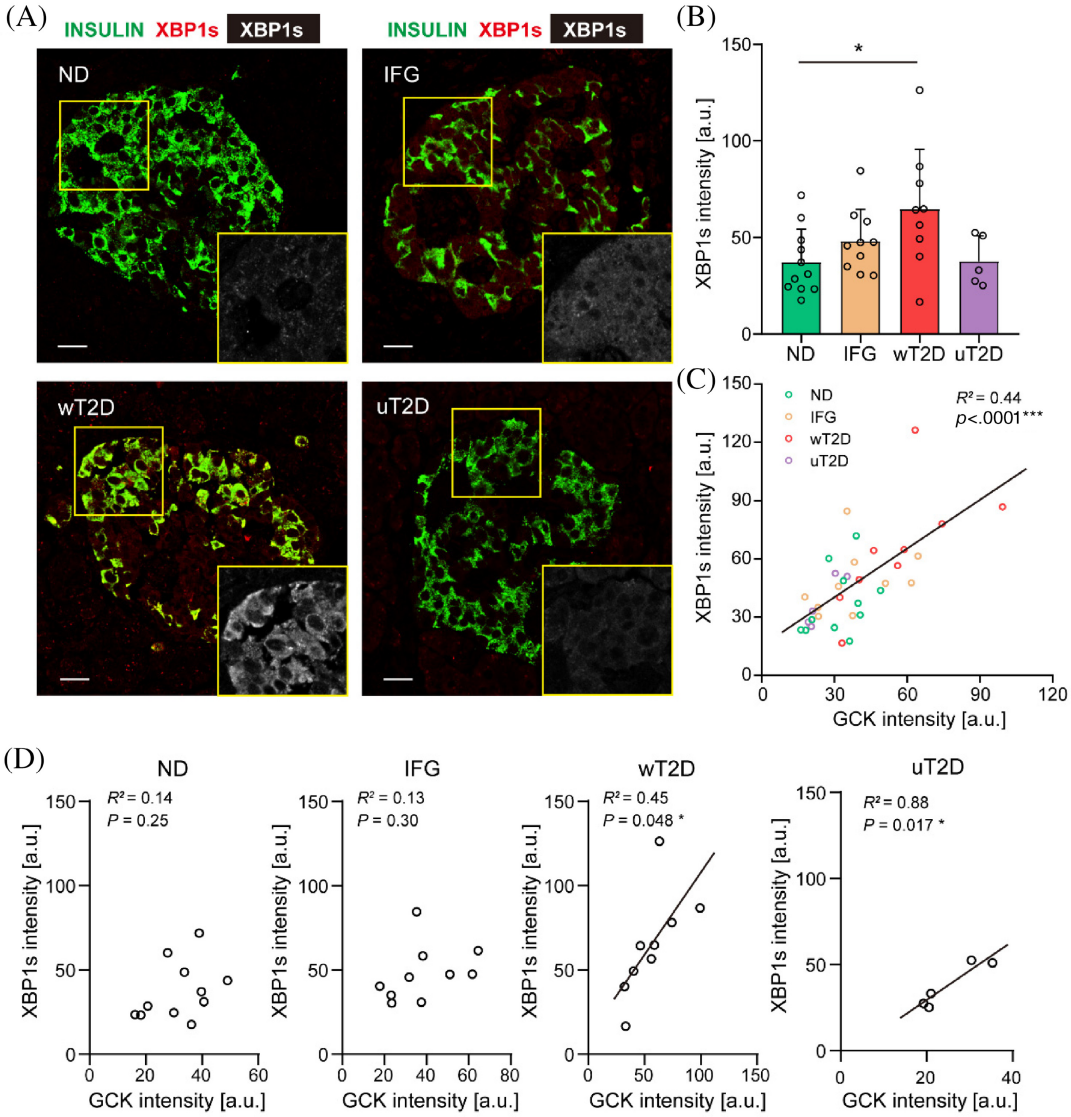
Quantificational and correlational analysis of XBP1s expression in human β‐cells. (A) Representative images of immunofluorescence staining for XBP1s (red and yellow box showing the XBP1s intensity in grayscale) and INSULIN (green). Scale bars, 20 μm. (B) Quantificational and statistical analysis of mean XBP1s intensity of β‐cells in ND (*n* = 11), IFG (*n* = 10), wT2D (*n* = 9), and uT2D (*n* = 5) individuals. (C, D) Linear regression analysis was performed to detect the relationship between GCK and XBP1s intensity in all individuals (C), or in ND, IFG, wT2D, and uT2D individuals, respectively (D). *R* ^2^ and *p* values are shown in each panel. Data presented as means ± SD. *p* less than 0.05 was considered statistically significant. *p < 0.05, ***p < 0.001. GCK, glucokinase; IFG, impaired fasting glucose; ND, nondiabetes; uT2D, uncontrolled type 2 diabetes; wT2D, well‐controlled type 2 diabetes; XBP1s, spliced X‐box binding protein 1.

**FIGURE 3:**
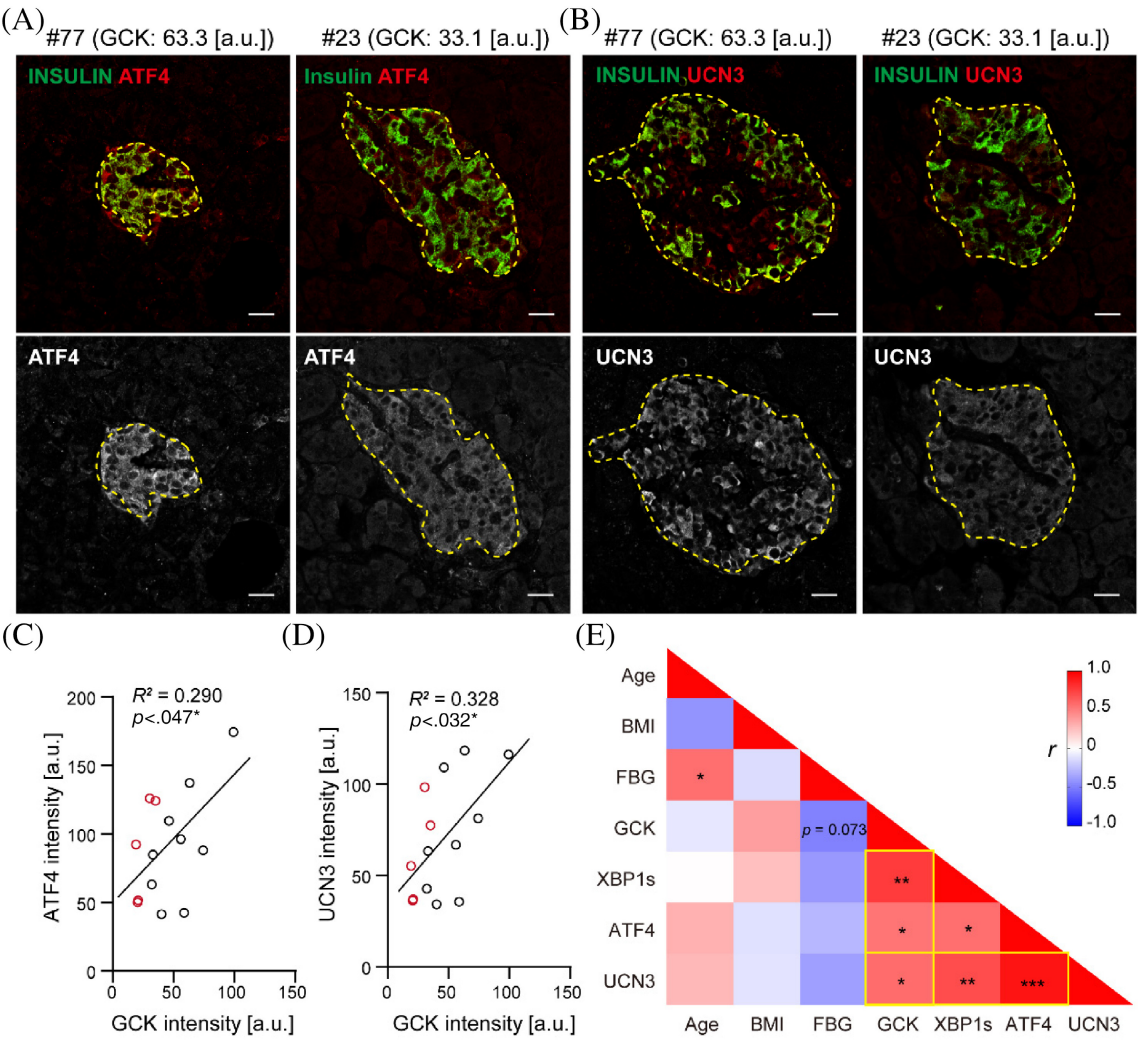
Activation of GCK induced increased expression of ATF4 and UCN3. (A, B) Representative images of immunofluorescence staining for ATF4 (A) or UCN3 (B) (red in merged channels and grayscale in split channel) and INSULIN (green) in individuals with high GCK expression (No.77, GCK: 63.3[a.u.]) and low GCK expression (No.23, GCK: 33.1[a.u.]). Scale bars, 20 μm. (C, D) Linear regression analysis was performed to detect the relationship between GCK and ATF4 (C) or UCN3 (D) expression in T2D. The black circles represent the individuals of wT2D, and the red circles represent uT2D. (E) Multivariate correlation analysis between clinical parameters (age, BMI, and FBG) and gene expressions (GCK, XBP1s, ATF4, and UCN3) in T2D. ATF4, activating transcription factor; BMI, body mass index; FBG, fasting blood glucose; GCK, glucokinase; UCN3, urocortin‐3; uT2D, uncontrolled type 2 diabetes; wT2D, well‐controlled type 2 diabetes; XBP1s, spliced X‐box binding protein 1.

It has been suggested that sustained activation of GCK might induce adaptive UPR.^12^, ^17^, ^21^ Therefore, we examined the expression of the specific marker of UPR, XBP1s^22^, ^23^ in human islets from all the subjects examined (Figure 2A). Indeed, we detected a significant increase of XBP1s expression in β‐cells from wT2D (64.7 ± 31.0 in wT2D vs 37.3 ± 17.1 in ND, *p* = 0.041, Figure 2B) that had elevated β‐cell GCK levels. On the contrary, there was no significant difference of XBP1s expression in β‐cells between uT2D and ND (47.5 ± 23.0 in uT2D vs 37.3 ± 17.1 in ND, *p* = 0.95, Figure 2B). Moreover, we found a highly significant association between XBP1s and GCK levels in β‐cells of all subjects (*R* ^2^ = 0.44, *p* < 0.0001, Figure 2C). This positive correlation between GCK and XBP1s expression was not detectable in the ND or IFG groups (*R* ^2^ = 0.14, *p* = 0.25 in ND and *R* ^2^ = 0.13, *p* = 0.30 in IFG, Figure 2D) but was present in T2D patients, in spite of their glycemic levels (*R* ^2^ = 0.45, *p* = 0.048 in wT2D and *R* ^2^ = 0.88, *p* = 0.017 in uT2D, Figure 2D). We also checked another adaptive UPR marker, activating transcription factor 4 (ATF4) expression in β‐cells from T2D subjects. Similarly, we found a significant and positive association between ATF4 and GCK expression in diabetic human β‐cell, regardless of their glycemic levels (Figure 3A,C). However, we did not detect correlation of statistical significance (Figure S3C, *R* ^2^ = 0.076, *p* = 0.34) in GADD34 and GCK expression of β‐cells. These data indicate that GCK activation in diabetic β‐cell is closely linked to adaptive UPR response.

### 3.4 A strong positive correlation between GCK and UCN3 expression in diabetic human β‐cells

To test the functional state of diabetic islets with different GCK levels, we carried out immunohistochemistry with β‐cell functional marker UCN3 together with INSULIN in T2D individuals. We found that UCN3 expression tended to be more abundant in diabetic subjects with higher β‐cell GCK expression (Figure 3B). There was a strong positive correlation between GCK and UCN3 expressions in diabetic human β‐cells (Figure 3B,C), indicating GCK high expressing β‐cells were functionally hyperactive populations.

Our multivariate correlation analysis based on the clinical parameters and in situ β‐cell markers in diabetic subjects were summarized in Figure 3E, which revealed that wT2D subjects tended to recruit more β‐cells into a state with higher GCK expression and functionally active (higher UCN3). These higher GCK expressing β‐cells were associated with increased adaptive UPR response (higher XBP1s and ATF4) to compensate for the increased metabolic influx (Figure 3E), thus maintaining normal blood glucose level compared to uT2D. On the contrary, defects in GCK activation in diabetic β‐cells with lower GCK expression showed inadequate adaptive UPR response and lower UCN3 expression and led to decompensation of β‐cells and poor glycemic control (*r* = −0.49, *p* = 0.073, Figure 3E). These data suggest that establishment of a proper coordination between GCK and adaptive UPR response is crucial in human β‐cell compensation during diabetes progression.

## 4 DISCUSSION

GCK is present in many organs of the human body, especially two key organs of glucose metabolism, pancreatic islet and liver.^24^ Emerging evidence suggests that GCK is responsible for coupling glucose metabolism to insulin secretion in β‐cells^25^, ^26^, ^27^, ^28^ and is required for the increase in β‐cell mass in high‐starch diet^29^ and high‐fat diet mice.^30^ During the past decades, GCK was identified as a new, promising drug target for T2D.^11^ Thus, numerous small‐molecule GKAs have been developed and clinically tested,^4^ some of them showing desirable effects on blood glucose‐lowering.^5^, ^7^ However, the effects of prolonged excessive glycolysis and induced β‐cell hyperactivation are still debated.^31^ Several studies showed that long‐term activation of GCK resulted in metabolic stress and glucotoxicity in β‐cells,^15^, ^16^ whereas reducing GCK activity enhanced insulin secretion and decreased metabolic stress‐induced genes in islets of db/db mice.^17^, ^21^ The involvement of human β‐cell GCK expression during pathophysiological progression of T2D is currently unknown.

Pancreatic β‐cells in T2D individuals have several defects,^32^, ^33^ including decreased β‐cell mass and impaired glucose‐stimulated insulin secretion.^34^, ^35^, ^36^, ^37^, ^38^ It has been reported that human β‐cells can adapt to metabolic stress by enhancing β‐cell function and number via mammalian target of rapamycin complex 1 signaling,^19^ glucagon‐like peptide‐2 receptor signaling,^39^ mitonchondrial activation,^40^ and the chemical serotonin (5‐hydroxytryptamine, 5‐HT) in pregnancy^41^ eventually lead to increasing insulin secretion to some extent. This is the first study exploring dynamic change of GCK expression in human pancreatic islets under different stages of T2D. We evaluated GCK expression in insulin^+^ or glucagon^+^ areas from pancreatic sections of 11 ND, 10 IFG, and 14 T2D. The β‐cell GCK expression was rather low in ND subjects, and no further increase was detected in IFG subjects. It is possible that GCK expression at the mRNA level is largely constitutive and stable in pancreatic β‐cells.^42^ Interestingly, we observed a significant upregulation of GCK expression in T2D with adequate glucose control, but such increase was not present in T2D with poor glycemic control. The negative association between FBG and GCK expression in diabetic β‐cells indicate that GCK activation may be required for successful β‐cell adaptation and adequate glycemic control. This was further confirmed by our finding that functional marker UCN3 was indeed increased in diabetic β‐cells with higher GCK expression, indicating these cells are functionally activated. The change in GCK expression in β‐cells was consistent with the observation in liver from T2D patients, in which hepatic GCK was also negatively correlated with fasting blood glucose.^43^

It has been reported that metabolic overload in β‐cell resulted in accumulation of misfolded protein and UPR induction.^44^, ^45^ The UPR is a critical adaptive response to ER stress serving to restore cell homeostasis when exceeding manageable levels.^23^ If the adaptive mechanism fails, the ER homeostasis is lost and ultimately results in β‐cell apoptosis^46^, ^47^ or identity loss.^45^ Defects in adaptive UPR have been reported in islets of ob/ob mice, highlighting the need for increased UPR signaling in β‐cell successful adaption.^48^ The inositol‐requiring transmembrane kinase/endoribonuclease 1α/XBP1s pathway is one of the UPR signaling factors that maintains ER homeostasis in the early adaptive phase of ER stress.^49^ In the present study, we observed a strong positive correlation between GCK levels and XBP1s expression in diabetic β‐cells. Moreover, we also found a positive correlation between expression levels of GCK and ATF4,^50^ another stress response transcription factor that is essential for β‐cell survival and identity under stress conditions^51^ in diabetic human β‐cells. These observations are relevant, because the adaptive mechanisms, such as adaptive UPR response, need to be stimulated in compensatory β‐cells with excessive glycolysis in order to cope with the high demand of insulin synthetic and secretory activity.^52^ These findings were consistent with a recent phase 3 clinical trial of novel dual‐acting GKAs, in which dorzagliatin achieved sustained glycemic control in the T2D participants with a relatively higher FBG (9.8 ± 1.8 mmol/L) baseline.^7^

Based on our novel observation on differences in β‐cell GCK expression between T2D patients with adequate and poor glycemic control, we proposed successful GCK activation in human β‐cells is beneficial for lowering blood glucose. Moreover, the fact that these GCK activated β‐cells with enhanced adaptive UPR response were functionally hyperactive indicated that GCK activation was one of the important adaptive mechanisms in T2D patients. Of course, we should note that our observations were gathered from T2D patients without GKA treatment; thus it cannot fully represent the β‐cell phenotype shift under long‐term GKA treatment. The exploration of GKAs requires in‐depth research and long‐term clinical trial follow‐ups.

## AUTHOR CONTRIBUTIONS

Jingwen Liu performed the experiments and analyzed the data. Hui Fu performed the experiments. Fuyun Kang performed the experiments. Qicheng Ni conceived and designed the experiments and wrote the manuscript. Qidi Wang wrote and reviewed the manuscript. Guang Ning and Weiqing Wang reviewed the manuscript. Qidi Wang is the guarantor of this work and, as such, had full access to all the data in the study and takes responsibility for the integrity of the data and the accuracy of the data analysis.

## FINANCIAL INFORMATION

This work was supported by the National Natural Science Foundation of China (81 870 527, 82 070 795, 82 100 835, 91 857 205, 81 730 023) and the Shanghai Sailing Program (21YF1426900).

## DISCLOSURE

The authors have nothing to disclose.

## ETHICS STATEMENT

This study was approved by the Institutional Review Board of the Ruijin Hospital affiliated to Shanghai Jiao Tong University School of Medicine and was in accordance with the principles of the Declaration of Helsinki.

## Supporting information


**Data S1.** Supporting Information

## Data Availability

All data generated or analyzed during this study are included in the article. Further information about the data are available from the corresponding author upon request.
