# Supplementary material for: β‐Cell glucokinase expression was increased in type 2 diabetes subjects with better glycemic control
Source: J Diabetes. 2023 Mar 20;15(5):409–18. doi: 10.1111/1753-0407.13380 (PMC10172022; doi:10.1111/1753-0407.13380)
Supplement: Supplementary file 1 — Data S1. Supporting Information [file JDB-15-409-s001.docx]

**Supplementary Information**

**β-cell glucokinase expression was increased in type 2 diabetes subjects with better glycemic control**

**Liu et al**


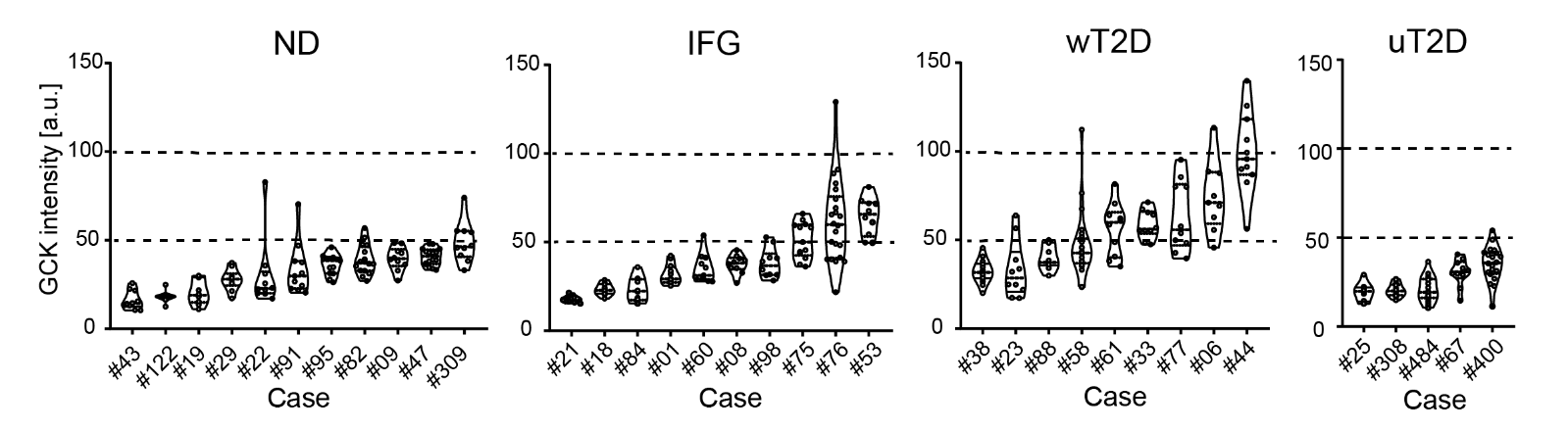


**Supplementary Fig. 1 Violin plots showed the GCK expression of each islet in ND, IFG, wT2D and uT2D individuals.**


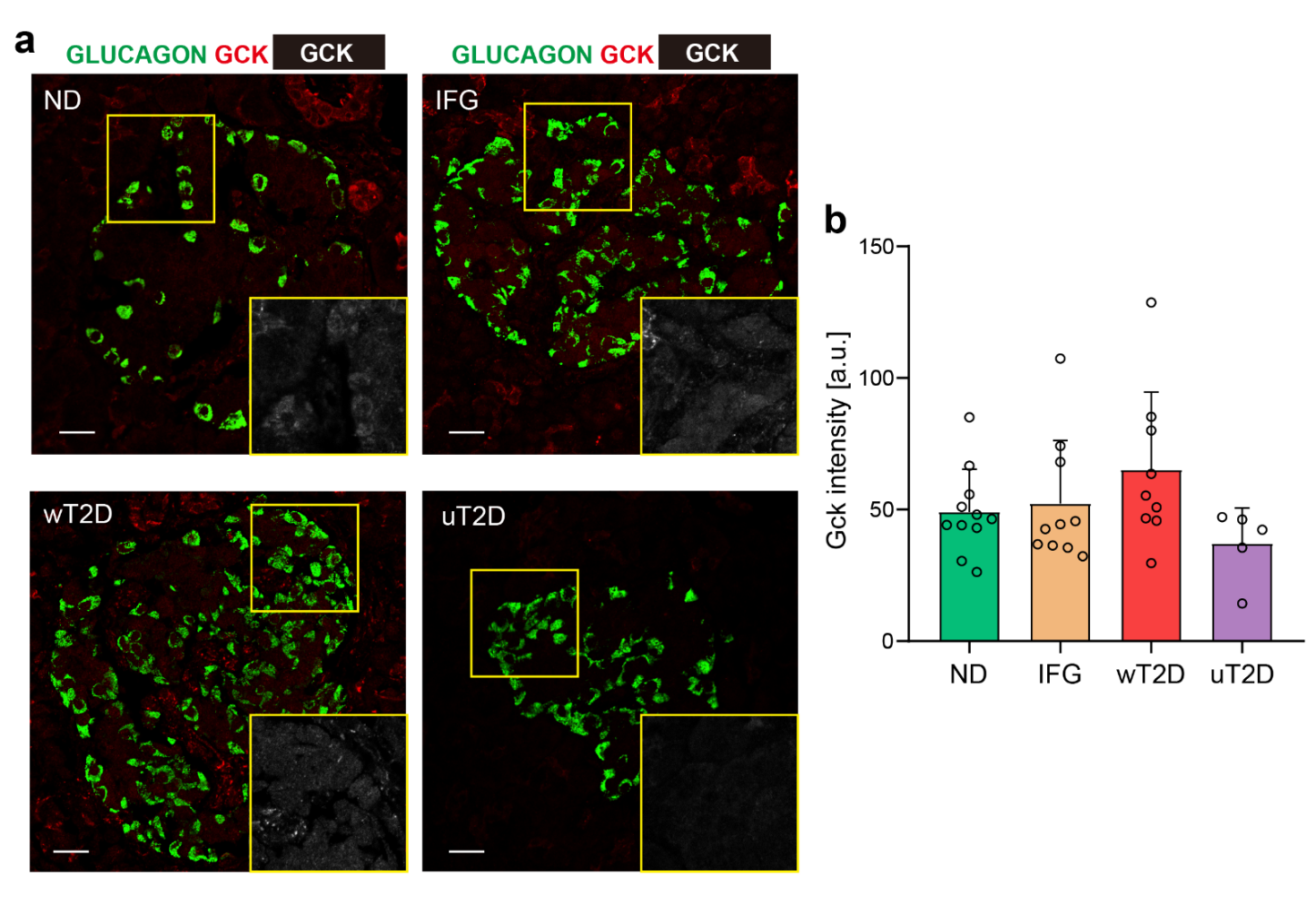


**Supplementary Fig. 2 Comparable GCK expression in α-cells among the four groups.** (**A**) Representative images of pancreatic sections stained for GCK (red in merged channels and grayscale in split channel) from nondiabetic, impaired fasting glucose, well controlled T2D and uncontrolled T2D individuals. Scale bars, 20μm. (**B**) Quantification and statistical analysis of mean GCK intensity of α-cells in ND (n = 11), IFG (n = 10), wT2D (n = 9) and uT2D (n = 5). Analysis of variance with Bonferroni post hoc test was performed between the 4 groups. *P* less than 0.05 was considered statistically significant.

**Supplementary Fig. 3**


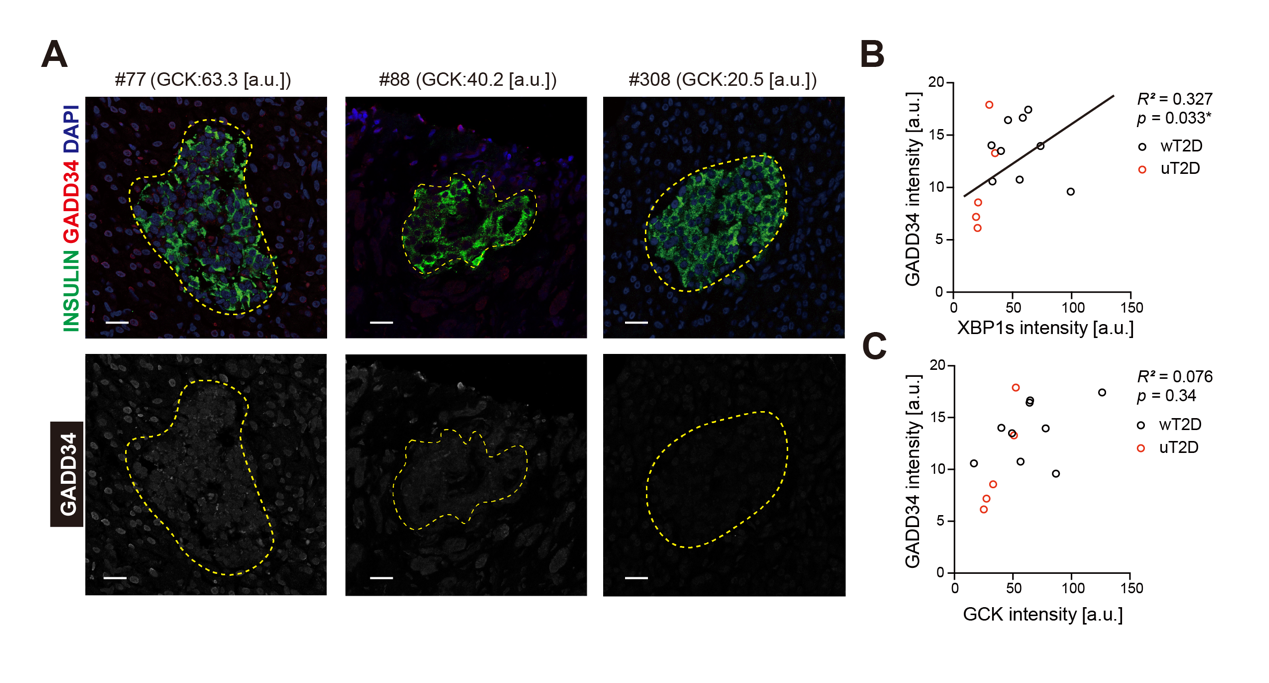


**Supplementary Fig. 3 Quantificational and correlational analysis of GADD34 expression in human β-cells.** (**A**) Representative images of immunofluorescence staining for GADD34 (red and yellow box showing the GADD34 intensity in grayscale) and INSULIN (green). Scale bars, 20μm. (**B, C**) Linear regression analysis was performed to detect the relationship between GADD34，GCK and XBP1s intensity in diabetic individuals. *R^2^* and *P* values are shown in each panel. *P* less than 0.05 was considered statistically significant. * *P* < 0.05.

**Table S1. Characteristics of ND, IFG and T2D individuals**

| Case ID | Sex | | Age (years) | BMI (kg/m^2^) | FBG (mmol/L) | Diagnosis | Treatment of diabetes | |
| --- | --- | --- | --- | --- | --- | --- | --- | --- |
| ND |  | |  |  |  |  | |  |
| #309 | F | | 70 | 19.61 | 5.47 | PSM | | / |
| #19 | M | | 64 | 23.03 | 4.58 | PSC | | / |
| #43 | F | | 25 | 22.19 | 5.12 | PSM | | / |
| #29 | M | | 56 | 20.66 | 4.95 | PMC | | / |
| #91 | F | | 71 | 22.73 | 4.50 | PMC | | / |
| #22 | M | | 58 | 24.62 | 5.12 | PSC | | / |
| #82 | F | | 39 | 20.81 | 4.68 | PMC | | / |
| #122 | M | | 53 | 18.60 | 5.21 | SPNP | | / |
| #47 | M | | 51 | 27.18 | 5.47 | SPNP | | / |
| #95 | F | | 56 | 23.72 | 5.00 | PMC | | / |
| #09 | M | | 35 | 21.60 | 5.23 | Other*^1^ | | / |
| Mean ± SD | | | 52.50 ± 14.4 | 22.3 ± 2.4 | 5.0 ± 0.3 | / | | / |
| IFG |  | |  |  |  |  | |  |
| #01 | M | | 68 | 25.93 | 6.02 | SPNP | | / |
| #21 | F | | 41 | 22.24 | 5.9 | SPNP | | / |
| #84 | F | | 54 | 22.52 | 5.93 | PMC | | / |
| #60 | F | | 56 | 22.66 | 5.79 | PMC | | / |
| #53 | M | | 54 | 25.71 | 5.66 | ECIPAS | | / |
| #08 | M | | 56 | 24.76 | 6.62 | PMC | | / |
| #18 | M | | 66 | 24.91 | 6.23 | PMC | | / |
| #76 | F | | 51 | 21.09 | 6.06 | PSC | | / |
| #98 | F | | 61 | 27.78 | 5.93 | PSM | | / |
| #75 | M | | 59 | 22.99 | 6.80 | PMC | | / |
| Mean ± SD /  Median(range) | | | 56.6 ± 7.7 | 24.1 ± 2.1 | 6.1±0.4 | / | | / |
| wT2D |  | |  |  |  |  | |  |
| #23 | F | | 69 | 18.83 | 5.00 | PSC | | Metformin |
| #33 | F | | 49 | 26.22 | 5.79 | PMC | | Acarbose+Insulin |
| #06 | M | | 64 | 28.54 | 6.75 | PMC | | Insulin+Metformin+ Glibenclamide |
| #88 | M | | 52 | 23.03 | 6.58 | PMC | | Insulin |
| #58 | F | | 54 | 23.44 | 5.22 | PSC | | Insulin |
| #44 | M | | 63 | 23.42 | 6.74 | PMC | | Acarbose+Insulin |
| #77 | F | | 69 | 21.26 | 5.78 | PMC | | Metformin |
| #61 | F | | 64 | 24.22 | 7.36 | PMC | | Insulin |
| #38 | F | | 46 | 24.24 | 5.80 | PSM | | Insulin+Metformin+Voglibose |
| Mean ± SD /  Median(range) | | | 58.9 ± 8.7 | 23.7 ± 2.8 | 6.1 ± 0.8 | / | | / |
| uT2D | |  |  |  |  |  | |  |
| #25 | | F | 56 | 23.23 | 8.15 | PMC | | Acarbose+Metformin+Insulin |
| #400 | | M | 65 | 25.65 | 8.24 | PMC | | Insulin |
| #308 | | F | 71 | 20.19 | 13.7 | IPMN | | Insulin |
| #67 | | M | 72 | 19.94 | 9.1 | IPMN | | Insulin |
| #484 | | M | 73 | 24.21 | 13.72 | Other*^2^ | | None |
| Mean ± SD /  Median(range) | | | 67.4±  7.1 | 22.6±  2.5 | 10.6±2.9 | / | | / |

All values are expressed as mean ± SD.

Abbreviation: M, male; F, female; BMI, body mass index; FBG, fasting blood glucose; PMC, pancreatic mucinous cystadenoma; PSC, pancreatic serous cystadenoma; PSM, pancreatic serous microcyst adenoma; SPNP, solid pseudopapillary neoplasm of the pancreas; IPMN, intraductal papillary mucinous neoplasm. *1, cirrhosis and refractory variceal bleeding. ECIPAS, Epidermoid cyst in intrapancreatic accessory spleen. *2, neoplasm of common bile duct epithelium

**Table S2. The number of islets evaluated for each analysis**

| Case ID | GCK on β cells | | GCK on ⍺ cells | XBP1s | ATF4 | GADD34 | | UCN3 | |  |
| --- | --- | --- | --- | --- | --- | --- | --- | --- | --- | --- |
| ND |  | |  |  |  |  | |  | |  |
| #309 | 10 | | 18 | 28 | / | / | | / | |  |
| #19 | 10 | | 11 | 11 | / | / | | / | |  |
| #43 | 10 | | 12 | 11 | / | / | | / | |  |
| #29 | 10 | | 11 | 10 | / | / | | / | |  |
| #91 | 13 | | 7 | 10 | / | / | | / | |  |
| #22 | 10 | | 14 | 20 | / | / | | / | |  |
| #82 | 16 | | 14 | 10 | / | / | | / | |  |
| #122 | 10 | | 10 | 8 | / | / | | / | |  |
| #47 | 16 | | 12 | 21 | / | / | | / | |  |
| #95 | 11 | | 9 | 10 | / | / | | / | |  |
| #09 | 10 | | 11 | 14 | / | / | | / | |  |
| Mean ± SD 11.5±5.9 | | | 11.7±8.4 | 13.9±39.9 | / | / | | / | |  |
| IFG |  | |  |  |  |  | |  | |  |
| #01 | 12 | | 23 | 26 | / | / | | / | |  |
| #21 | 10 | | 19 | 10 | / | / | | / | |  |
| #84 | 10 | | 8 | 6 | / | / | | / | |  |
| #60 | 11 | | 10 | 10 | / | / | | / | |  |
| #53 | 10 | | 16 | 12 | / | / | | / | |  |
| #08 | 10 | | 16 | 17 | / | / | | / | |  |
| #18 | 11 | | 14 | 17 | / | / | | / | |  |
| #76 | 22 | | 16 | 10 | / | / | | / | |  |
| #98 | 10 | | 10 | 10 | / | / | | / | |  |
| #75 | 13 | | 14 | 14 | / | / | | / | |  |
| Mean ± SD 11.9±13.7 | | | 14.6±20.3 | 13.2±32.0 | / | / | | / | |  |
| wT2D |  | |  |  |  |  | |  | |  |
| #23 | 10 | | 18 | 11 | 10 | 11 | | 10 | |  |
| #33 | 10 | | 18 | 10 | 10 | 11 | | 10 | |  |
| #06 | 9 | | 9 | 12 | 10 | 10 | | 10 | |  |
| #88 | 10 | | 16 | 15 | 10 | 13 | | 10 | |  |
| #58 | 25 | | 12 | 10 | 10 | 10 | | 10 | |  |
| #44 | 11 | | 10 | 11 | 10 | 10 | | 10 | |  |
| #77 | 10 | | 11 | 11 | 10 | 10 | | 10 | |  |
| #61 | 10 | | 12 | 18 | 10 | 11 | | 10 | |  |
| #38 | 17 | | 15 | 10 | 19 | 11 | | 10 | |  |
| Mean ± SD 12.4±27.8 | | | 13.4±11.5 | 13.2±7.5 | 11.0±9.0 | 10.8±0.9 | | 10.0±0.0 | |  |
| uT2D | |  |  |  |  |  | |  | |  |
| #25 | | 10 | 9 | 17 | 10 | 12 | | 10 | |  |
| #400 | | 20 | 13 | 17 | 10 | 10 | | 10 | |  |
| #308 | | 21 | 18 | 18 | 16 | 11 | | 10 | |  |
| #67 | | 10 | 14 | 9 | 9 | 10 | | 10 | |  |
| #484 | | 23 | 10 | 17 | 10 | 11 | | 10 | |  |
| Mean ± SD 16.8±39.7 | | | 12.8±12.7 | 12.0±13.8 | 11.0±8.0 | | 10.8±0.7 | | 10.0±0.0 | |

All values are expressed as mean ± SD.
